# A Clinical TB Detection Method Based on Molecular Typing Technique with Quality Control

**DOI:** 10.1155/2019/9872425

**Published:** 2019-03-25

**Authors:** Tienan Feng, Yan Cheng, Suwen Yu, Feng Jiang, Min Su, Jin Chen

**Affiliations:** ^1^Hongqiao International Institute of Medicine, Shanghai Tongren Hospital, Shanghai Jiao Tong University School of Medicine, Shanghai 200335, Shanghai, China; ^2^Clinical Research Center, Shanghai Jiao Tong University School of Medicine, Shanghai 200025, Shanghai, China; ^3^Department of Neurology, People's Liberation Army 102 Hospital, Changzhou 213003, Jiangsu, China; ^4^Dapuqiao Community Health Service Center, Shanghai 200025, Shanghai, China; ^5^Department of Laboratory, First Affiliated Hospital of Hunan University of Chinese Traditional Medicine, Changsha 410007, Hunan, China; ^6^Department of Laboratory, First People's Hospital, Lianyungang 222002, Jiangsu, China; ^7^Clinic and Research Center of Tuberculosis, Shanghai Key Lab of Tuberculosis, Shanghai Pulmonary Hospital, Shanghai 200433, Shanghai, China

## Abstract

The gold standard for diagnosing pulmonary *Mycobacterium tuberculosis* (TB) is the detection of tubercle bacillus in patient sputum samples. However, current methods either require long waiting times to culture the bacteria or have a risk of getting false-positive results due to cross-contamination. In this study, a method to detect tubercle bacillus based on the molecular typing technique is presented. This method can detect genetic units, variable number of tandem repeat (VNTR), which are the characteristic of tuberculosis (TB), and performs quality control using a mathematical model, ensuring the reliability of the results. Compared to other methods, the proposed method was able to process and diagnose a large volume of samples in a run time of six hours, with high sensitivity and specificity. Our method is also in the pipeline for implementation in clinical testing. Reliable and confirmed results are stored into a database, and these data are used to further refine the model. As the volume of data processed from reliable samples increases, the diagnostic power of the model improves. In addition to improving the quality control scheme, the collected data can be also used to support other TB research, such as that regarding the evolution of the tubercle bacillus.

## 1. Introduction

In 2016, there were an estimated 10.4 million (95% uncertainty interval 8·8–12·2) new incident cases of *Mycobacterium tuberculosis* (MTB) worldwide. While the rate of increase of tuberculosis (TB) incidence has slowed down since 2005, the population with latent and asymptomatic TB continues to grow, particularly in developing countries [1]. Given the potential complications of untreated TB, early and accurate diagnosis after the onset of symptoms can result in a significant improvement in the outcome [2, 3]. However, it is widely known that current diagnostic methods are time-consuming or not accurate enough, limiting progress in the prevention and control of TB-related diseases [4].

The gold standard for TB diagnosis is finding MTB in clinical samples of the patient that include sputum or other specimen [5]. The smear test of the primary specimen is the usual method, while the most reliable method of confirming the presence of the tubercle bacillus in the patient's specimen is culture. However, this requires a waiting period of 3 to 6 weeks [6, 7], which results in uncertain treatment status for suspected TB patients during this long process of diagnosis. TB bacteria can also be difficult to cultivate, leading to low sensitivity of smear microscopy [8, 9]. Other methods, such as polymerase chain reaction (PCR) and the immunological ones, require less time but may generate false-positive results due to cross-contamination [10]. Therefore, it is important for clinicians, before making a definitive diagnosis, to take into consideration all the information, including X-ray imaging, clinical symptoms, therapeutic effects, and clinical history. The consistency of information helps us to improve the reliability of diagnosis. Thus, research on a TB diagnostic approach able to combine rapidity and accuracy is still ongoing [11–13].

In the present study, in order to overcome these two technical difficulties in TB detection, a method based on a molecular typing technique and mathematical modeling was proposed. Molecular typing methods based on variable number tandem repeat (VNTR) analysis have been used to identify MTB in epidemiologic studies [14–17]. VNTR sequences have emerged as valuable markers for the genotyping of several bacterial species, especially of genetically homogeneous pathogens, such as *Bacillus anthracis*, *Yersinia pestis,* and *M. tuberculosis* complex members. Initial VNTR typing systems for MTB complex strains made use of very limited sets of loci, which turned out to be not discriminatory enough. Subsequently, more extensive sets of VNTR loci have been described, including a system based on 12 loci, which has been shown to be applicable for reliable genotyping and molecular epidemiology studies of *M. tuberculosis*. These loci contain VNTR of genetic elements named mycobacterial interspersed repetitive units (MIRUs), which are located mainly in intergenic regions dispersed throughout the *M. tuberculosis* genome. All above loci are collectively designated as MIRU-VNTR loci [18]. Due to their ease of operation, high sensitivity, and specificity, they have the potential to be used as an MTB detection method. In the present study, the detection of MTB was focused on the MTB genotypes based on MIRU*-*VNTRs in regions dispersed throughout the MTB genome [19]. However, false positives from cross-contamination, pollution of the nucleic acid amplification test (NAAT) reagent, and their exacerbation by nucleic acid amplification remain inevitable and unidentifiable. Despite the fact that the rate of contamination may be low, any contamination that does occur is amplified and can lead to serious clinical consequences. Therefore, a stringently designed quality control (QC) scheme is essential to guarantee high testing accuracy before a testing method can be brought into clinical use [20, 21]. In current practice, before testing the patient samples, clinical technicians must calibrate their equipment using standard samples. However, this still cannot eliminate confounding factors, such as cross-contamination, since traditional QC strategies only evaluate the reliability of reagents and equipment [22–24]. This is the reason why molecular amplification techniques lack a method for ensuring the reliability of samples during the testing process, resulting in diagnostic uncertainty and, in the worst case, in misdiagnosis. Mathematical methods have been used in medicine for many decades. In this study, a mathematical QC model for molecular amplification technique based on binomial distribution was developed, which addresses potential contamination issues caused by the technique. In order to evaluate the proposed model, real TB samples were used.

## 2. Materials and Methods

### 2.1. Samples

516 clinical sputum specimens were collected from the Shanghai Pulmonary Hospital of Tongji University between February and October 2014. Among them, 9 were excluded due to uncertain diagnoses, and 13 were removed since they were obtained from cured patients whose TB results were affected by drugs. Form the remaining 494 samples, 167 were confirmed as TB and 327 were found to be non-TB, including 4 samples of nontuberculous mycobacteria (NTM). The diagnosis of the TB cases was also based on X-ray imaging, clinical symptoms, therapeutic effects, and clinical history. Finally, 148 samples with complete data sheets and confirmed diagnosis were included in the study. The prior distribution of different subtypes of TB bacteria was calculated based on their VNTR results.

### 2.2. MIRU-VNTR Method

In the proposed method, the aforementioned MIRU-VNTR loci were used as the characteristics of different TB subtypes [25], and more specifically, MTUB21, MTUB04, QUB-18, QUB-26, QUB-11b, MIRU31, MIRU10, and MIRU26 were used due to the fact that they can be amplified at the same temperature. The repeat counts for each locus were set as the identifiers of the corresponding sample in the form of a numerical array. For example 1, 2, 3, 4, 5, 6, 7, and 8 represented a TB sample whose repeat counts for MTUB21, MTUB04, QUB-18, QUB-26, QUB-11b, MIRU31, MIRU10, and MIRU26 were 1, 2, 3, 4, 5, 6, 7, and 8, respectively. In theory, the occurrence rate of any given array is 1/10^8^, assuming even distribution of each locus with a maximum repeat count of 10. The frequency of repeat counts for each locus can be measured from the sample data. Then, as it can be seen in Table 1, the prior probability of each array can be calculated from the total number of permutations, following formula (1). In the QC step, the following scheme was used: if the expected occurrence rate is greater than the cutoff value, the testing results can be used to support the clinical practice:

(1) $$\begin{array}{c} P\left(2,3,4,4,5,8,6,4\right)=e^{\text{Ln}O_{1,2}+\text{Ln}O_{2,3}+\text{Ln}O_{3,4}+\text{Ln}O_{4,4}+\text{Ln}O_{5,5}+\text{Ln}O_{1,2}+\text{Ln}O_{6,8}+\text{Ln}O_{7,6}+\text{Ln}O_{8,4}}, \end{array}$$

where *O*_*i*,*j*_ is the occurrence count of one locus, *i* is the column number of Table 1, representing the type of loci, and *j* is the number of repeat counts.

### 2.3. Quality Control Model

MIRU-VNTR helps in extracting features of TB, and the chosen loci can be analyzed with PCR (a biological technique able to amplify the signal of such features) in the same temperature, in order to obtain a clear signal. Therefore, features of TB can be extracted with less time and cost. However, the contamination can also be amplified during the PCR process. In order to address this problem, it is important to know whether the contamination is reasonable. Based on the prior distribution of each locus, the occurrence rate of different TB subtypes was calculated. The occurrence rate of one TB subtype in a batch of samples conforms to the law of binomial distribution. Subsequently, using the binomial distribution theory, one could evaluate whether the contamination is reasonable.

Each sample had a specific numerical array and its corresponding prior distribution. As the occurrence probability of any given array is very low, the probability of an array appearing more than once in a single batch is far lower. Knowing the sample number in the testing batch and the prior distribution of each array, the expected occurrence rate (EOR) of an array can be calculated based on the binomial distribution function using the following formula in the event of two repeated loci:

(2) $$\begin{array}{c} \text{ES}=1-c_{n}^{1}p^{1}{\left(1-p\right)}^{n-1}-{\left(1-p\right)}^{n}, \end{array}$$

and the following (general) formula in the event of three or more:

(3) $$\begin{array}{c} \text{ES}=1-\overset{m}{\underset{c=2}{\sum }}c_{n}^{c-1}p^{c-1}{\left(1-p\right)}^{n-c+1}-{\left(1-p\right)}^{n}, \end{array}$$

where *C* is the permutation operation, *p* is the prior probability of the relevant array, *n* is the number of samples in the batch, and *m* is the number of copies. The value of ES is the expected occurrence rate for multiple copies of that array. In general, the cutoff value was set as 0.05. The arrays with an ES greater than 0.05 are thought to be so rare that if two or more copies of these appear in a batch, the result should be considered dubious. For arrays with an ES between 0.05 and 0.1, a result of multiple copies in a single batch should be cross-referenced against the patient's clinical information. For an array with an ES greater than 0.1, multiple copies are considered as a reasonable result. When the model was a multiple testing problem, the corrected cutoff value (cv), calculated by 1 − (1 − cv)^*n*^ = 0.05, was used to evaluate the results. Samples qualified as valid and reliable were stored in a database, whose data were used to derive prior distributions. As the sample size increased, the prior distribution approached that of the actual population, further improving the practicality and accuracy of diagnosis. Consequently, besides its utility in specimen testing, the proposed scheme is also able to improve research on the epidemiology of TB.

### 2.4. Ethical Considerations

Community surveys were conducted and approved by the ethical committees of the Shanghai Pulmonary Hospital of Tongji University. No human tissue was used in this study. Two of the coauthors of the paper provided clinical documents and clinical sputum specimens. The collected data included conformed diagnosis results and MIRU-VNTR results from the sputum specimens. No private information was used in this study. The data were used only for research purposes. The content of the study was written in the informed consent form, which was signed by all patients. All acquired records and specimens used in the study were anonymized and could not be linked to any of the patients. The ethical committees of the Shanghai Pulmonary Hospital of Tongji University approved all the experimental protocols. The methods carried out in this study were in accordance with the approved guidelines.

## 3. Results and Discussion

### 3.1. Prior Distribution of Two Test Results

A total of 148 samples confirmed as TB was collected over a two-month period, of which 92 were collected in the first month and 76 in the second.  Table 2 shows the prior distribution of each MIRU-VNTR locus. The distribution was not even, similar to previously reported results [26]. The occurrence rate of 3 repeated MIRU10 (3-MIRU10) loci was in both batches, the highest at 76.1%. The full high-occurrence list based on all data (148 samples) (>0.1), included 4-, 5-MTUB21; 3-, 4-, 5-MTUB04; 8-, 9-, 10-QUB-18; 7-, 8-, 9-QUB-26; 4-, 5-, 6-, 7-QUB-11b; 5-MIRU31; 2-, 3-MIRU10; and 8-, 9-MIRU26 loci. Most arrays demonstrated little difference (calculated as the result of the sample collected in the first month minus the result of the sample collected in the second) between the two collections (<0.05), with the exception of 3-MTUB04, 4-MTUB04, 4-QUB-26, and 8-QUB-11b.

### 3.2. Classification of TB Based on MIRU-VNTR

The occurrence rate of each strain of tubercle bacillus was calculated using the equation Ln(*P*_array_)=*∑*_*i*=1_^8^Ln(*O*_*ij*_) based on the results of Table 2. The theoretical number of possible arrays was 13^8^–1, with “−1” accounting for the (0, 0, 0, 0, 0, 0, 0, 0) array, which corresponded to (0-MTUB21, 0-MTUB04, 0-QUB-18, 0-QUB-26, 0-QUB-11b, 0-MIRU31, 0-MIRU10, 0-MIRU26), and it indicated that no tuberculosis was detected. The allowable permutations from Table 1 resulted to 9 *∗* 5 *∗* 13 *∗* 10 *∗* 8 *∗* 9 *∗* 6 *∗* 10 − 1 = 25,272,000 − 1 arrays in total. Among all arrays, the highest occurrence 0.005 was found for the array corresponding to the subtypes 5-MTUB21, 4-MTUB04, 8-QUB-18, 8-QUB-26, 6-QUB-11b, 5-MIRU31, 3-MIRU10, and 8-MIRU26.

**Figure 1:**
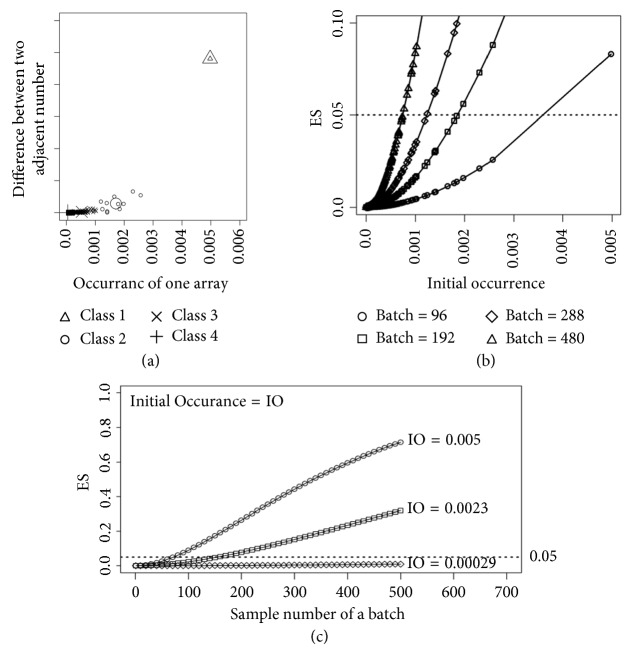
Distribution of VNTR assays. (a) The occurrence of high-frequency array: the higher the occurrence of the arrays, the rarer they are. (b) The reasonable repetition for batches of different sample numbers: repetition in a single batch is highly improbable for most TB subtypes. (c) The change of expected occurrence along with positive sample number in one batch: the occurrence rate of a given array may be very low, but the likelihood of it occurring twice or more increases as the number of positive samples rises.

MIRU-VNTR assays were classified as high frequency if the repeat distribution of any of its loci exceeded 0.1. This category comprised 864 TB subtypes, with occurrences ranging from 0.005 to 5.07*e*^−7^. After sorting the arrays in the descending order, it was found that the difference in occurrence between two contiguous arrays decreased as the occurrence decreased (Figure 1(a)), which means that high-occurrence arrays were rare and the occurrence of most arrays was very low. The K-means method was used to divide the 864 high-frequency arrays into 4 groups. Group 1 included only the array with the highest occurrence of 0.005. Group 2 contained 11 arrays with occurrence rates ranging from 0.001 to 0.0023. The vast majority of arrays fell into groups 3 and 4, ranging from 0.00029 to 0.001 and from 5.07*e*^−7^ to 0.00029, respectively.

The EOR of an array increased with the number of positive samples in the batch (Figure 1(c)). Each array has a possibility of occurring (e.g., 0.00029). Despite that the occurrence rate of a given array may be very low, the possibility of it occurring twice or more times increased as the number of positive samples increased. If the rate of an array that occurred twice or more times increased above the cutoff value of 0.05, the event was no longer considered as low rate. At this point, repeated occurrences of the array in a single batch were considered statistically reasonable. According to the results presented in Figure 1(b), a TB subtype with an occurrence rate of 0.0007 could reasonably appear twice in a batch containing at least 480 positive samples, whereas subtypes with larger initial occurrence rates could reasonably be repeated in smaller batches. Nevertheless, repetition in a single batch was highly improbable for most TB subtypes. Considering the possibility of contamination, it was necessary to set a time window within which all samples were considered as a single batch. It was calculated that, out of a total of (25,272,000 − 1) TB subtypes, there were no more than 200 types of TB with a reasonable possibility of appearing twice.

The results of the parts are shown in Figure 1(a). In Figure 1(a), the 864 TB subtypes with the high-occurrence rate were classified into four groups, based on the distance between two adjacent numbers, which indicated that the occurrence rate of most TB subtypes was very low. In Figure 1(c), three TB subtypes were chosen, with initial occurrences (IOs) 0.005, 0.0023, and 0.00029. This suggested that the *p* value (occurrence rate) increased as the sample number increased. In Figure 1(b), the relationship between ES and IO is demonstrated. If a batch contained more samples, higher occurrence of a TB subtype was reasonable, even if the IO of the subtype was low.

### 3.3. Simulation of the Process

**Figure 2:**
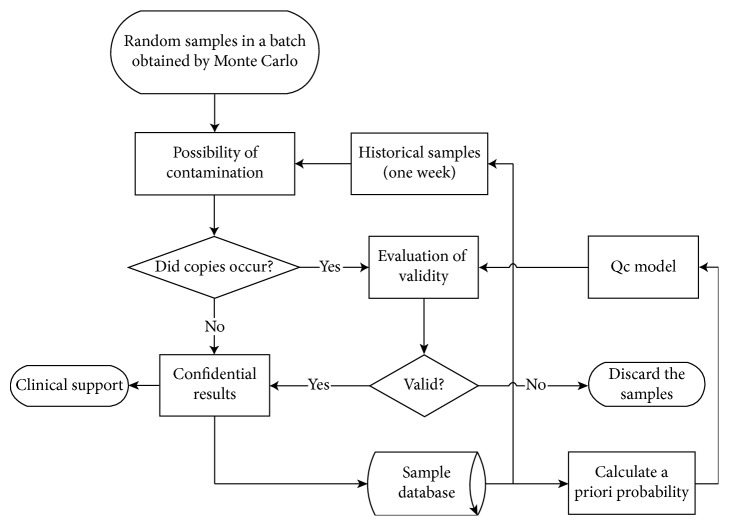
Simulating process using Monte Carlo.

In this subsection, a Monte Carlo algorithm was used to simulate the application of the proposed method in a clinical laboratory. The steps of this procedure are illustrated in Figure 2. In the first step, the random samples corresponding to one strain of tuberculosis were artificially generated. In this clinical test scheme, contamination was mainly simulated as cross-contamination due to airborne MTB, careless operation by staff, or contamination of equipment or reagents. As our proposed method entails the use of PCR amplification, any amount of cross-contamination could seriously affect the results. Instances of contamination were assumed to be random, low-frequency events, and that the contamination effects endure for some period of time. Therefore, the samples tested over a span of 1 week were considered part of the same batch and were used to calculate the expected occurrence rate of a strain of tuberculosis. When a repeated result occurred, we evaluated whether the repetition was reasonable based on if its expected occurrence rate was lower than the corrected cutoff value of 0.05, and the result was assumed to be contaminated and was ruled ineligible for use in clinical diagnosis. If the tested result succeeded the validity evaluation, it was also considered valid for clinical use, and the array was added to the QC database, where the prior distribution was recalculated, further refining the accuracy of diagnosis.

**Figure 3:**
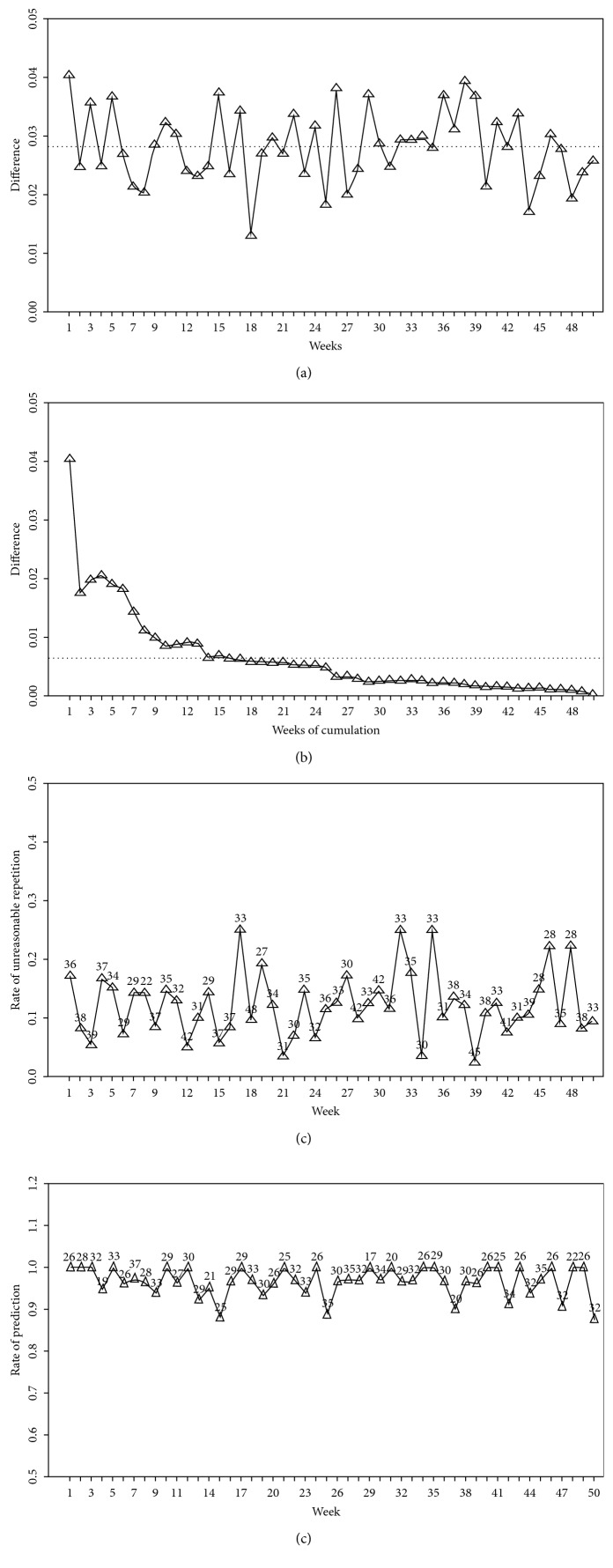
Results of simulation. (a) The result of the maximal difference between the distribution of all collected data and the distribution of data collected in each week in each week: these differences are small. (b) The result of the sum of absolute different values: the sum of absolute different values decreased as the accumulation of sample counts increased. (c) The rate of unreasonable repetition: the unreasonable repeated sample rate per week ranged from 0.05 to 0.25. (d) The accuracy of detection of contaminated samples: the accuracy ranged from 0.88 to 1.0.

The simulation time was set as 50 weeks, with an average of 1000 positive samples per simulation. The simulation results are demonstrated in Figure 3. From Figure 3(a), we know the maximum difference (max|*d*_*i*_^*E*^ − *d*_*i*_^*T*^|) between the distribution of all collected data and the distribution of data collected each week is disturbed, where *d*_*i*_^*E*^ is the distribution of each locus based on sample data in one week and *d*_*i*_^*T*^ is the distribution of each locus based on all collected sample data. The value of the sum of the absolute difference (sum|*d*_*i*_^*T*^ − *d*_*i*_^*E*^|) decreased as the accumulation of counts increased (Figure 3(b)), indicating that more data were collected and more accurate and thorough knowledge of the actual TB distribution was gained. For the first week, in order to construct the model, the available data of 1000 samples without cross-contamination were used. Since the model was a multiple testing problem, the corrected cutoff value (cv) was used to evaluate the results. In the model without the contamination function, 35 repeated samples were found. Among those, 29 had expected occurrence rate values larger than the cv and were considered as reasonable results that could be used in patient diagnosis, while 6 had expected occurrence rates below the cv, and thus, they were considered as invalid and were discarded.  Figure 3(c) shows the rate of nonreasonable repetition across all 50 weeks, with the total repeated sample number per week noted at the corresponding positions. The repeated sample numbers ranged from 22 to 45.

Data tested with the contamination function produced an average of 28.82 known contaminated samples each week, ranging from 17 to 38. In Figure 3(d), the rate of detection of contaminated samples *(contaminated samples detected by the model/total number of contaminated samples)* is shown, which ranged from 0.88 to 1.0. Further analysis showed that incorrect judgments made by the model were predisposed to contamination by high-occurrence samples, as well as false positives, when there was indeed a repetition of samples with low-occurrence rates. Based on the known contaminated condition of each sample, the proposed method demonstrated a sensitivity of 88.6% and a specificity of 98.14% in determining whether a sample is contaminated or not. The sensitivity was the rate of finding the contaminated samples that were preset, and the specificity was the rate of distinguishing the noncontaminated samples.

In Figure 3(a), the maximum difference between the distribution of each locus based on sample data of one week and the distribution of each locus based on all collected sample data was disturbed, indicating that the distribution of each locus calculated based on a date in a time window was not stable. As the accumulation of sample counts increased, the sum of the absolute differences decreased (Figure 3(b)), suggesting that the more the collected data, the more accurate and thorough the gained knowledge regarding the actual TB distribution. In Figure 3(c), the rate of unreasonable repetition is demonstrated, while in Figure 3(d), the accuracy of detection of contaminated samples is displayed, and it can be seen that the proposed model was able to detect the unreasonable repetition with a high accuracy.

When the proposed method was used on 148 TB cases, three repetitions were found. Due to their low-occurrence rate, the method assumed that the samples were contaminated, making a wrong diagnosis. The negative samples without contamination were expected to have no MIRU-VNTR features of TB. There was a very low probability that a sample may have a TB type with none of the MIRU-VNTR loci included in the study, which would bring a misdiagnosis. In our dataset, no patient provided TB specimens without at least one of the MIRU-VNTR loci included in the study based on the 327 confirmed TB-negative cases.

Despite the fact that there were some MIRU-VNTR loci whose distributions differed between the two tested results, most of these differences were negligibly small. In the simulation, the difference between the distribution of all collected data and that of the data collected during each week gradually decreased as the total accumulation of collected data in our database increased. The results suggested that most TB bacteria do not mutate frequently. Additionally, it was found that the TB subtypes (5-MTUB21, 4-MTUB04, 8-QUB-18, 8-QUB-26, 6-QUB-11b, 5-MIRU31, 3-MIRU10, and 8-MIRU26) were by far the most dominant subtype in the Shanghai area, with an appearance rate about 17 times that of the next most common subtype. Even though the exact reason why these loci remain stable has not been yet discussed, there is clinical significance in studying the effects (e.g., drug resistance) conferred by different types of tuberculosis. With the increasing accumulation of clinical information from patients, our database may contribute in making key inroads in this area of TB research.

Even small amounts of contamination, such as those obtained through aerosol, can be significantly amplified by PCR. Therefore, a longer time window of seven days was set for the tests. Based on the results, it was concluded that most arrays have an extremely low possibility of appearing twice within a time window. A laboratory that can confirm the elimination of some forms of contamination can set an even shorter time window. In this study, it was found that the high-frequency loci have an inordinately large influence on the testing results. If such results were involved in cross-contamination, the contamination may have been mistaken for a normal result. It was considered reasonable for the samples with an occurrence rate >0.0003 to have two or more copies in a single time window containing 1000 positive samples. These samples were classified into group(s) one (1), two (11), or three (61). The presence of more than two copies of some loci was also reasonable in this system. The most dominant subtype in the present study (5, 4, 8, 8, 6, 5, 3, 8) occurred 233 times and was almost consistent with its occurrence rate of 0.0005. Overall, the suggested testing method can account for a large majority of contamination incidents. However, if high-frequency loci occur twice or more in reasonable tested results, additional confirmation should be performed, such as thorough review of the patient's medical records.

Theoretically, the possibility that two strains of TB in the same clinical laboratory will have the same genetic features, namely, the same MIRU-VNTR loci repeats, is extremely low. If this happens, it is very plausible that one strain has contaminated the other. Based on this hypothesis, a rapid and accurate clinical scheme for TB testing was developed. In the clinical laboratory, eight samples were measured for eight to ten genetic sites at once and the repeat numbers of each genetic site were recorded as the special identifier of that subtype of TB. According to the results, the highest occurrence rate for any subtype appearing multiple times was 0.005 and was that of 5-MTUB21, 4-MTUB04, 8-QUB-18, 8-QUB-26, 6-QUB-11b, 5-MIRU31, 3-MIRU10, and 8-MIRU26. Despite the fact that this rate was already very low, it still dwarfs those of other subtypes. If a sample with a low-repetition rate appears twice or more, there is a high risk of contamination being the cause. Intracontamination is generally caused by poor procedures followed by the operating technician. When this happens, the suspect samples are recollected and retested. Intercontamination can be derived from contact between patients or airborne tuberculosis in the laboratory. In order to identify and solve these problems, the repeated results, the activity range of the corresponding patients, and the appearance and duration of symptoms should be investigated, all of which are included in the proposed method. If factors stemming from intra- and intercontamination can be excluded, but repeated samples with low-occurrence rates are still detected, it can be speculated that a subtype of TB may be creating an epidemic.

**Figure 4:**
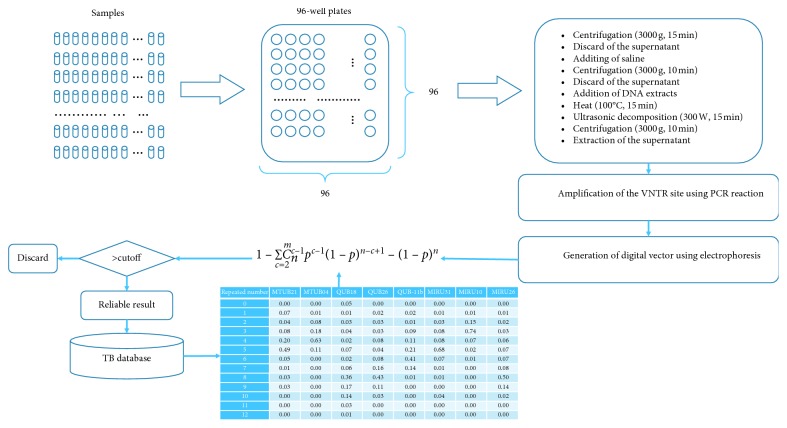
Scheme flowchart based on our model.

Compared to traditional TB testing, the proposed MIRU-VNTR method in this study can process large amounts of samples in very short time (Figure 4). Apart from the sputum specimen collected from patients, each step can be automated in a testing device and seamlessly connected in sequence. To account for potential cross-contamination of patient samples, the developed QC model not only can find a TB-positive patient with high accuracy but also can evaluate the reliability of the testing results.

## 4. Conclusions

In the present study, a method that can be widely used in epidemiological studies was proposed. However, due to the limited available volume of data, our method was insufficient to derive bias-free results. Based on the proposed testing scheme, digital MIRU-VNTR data extracted from collected sputum specimens can be automatically and constantly uploaded by clinicians using this scheme, allowing the analysis of large volumes of data and the acquisition of comprehensive and objective results. The data processing power of this technique may also aid researchers in determining the relationships between different TB subtypes and their clinical features, such as drug resistance [27], with the end goal of providing more accurate and personalized treatments. Apart from TB applications, this method can be also used in the detection of other viral diseases with genetic features, such as HIV and HBV. The proposed scheme was designed for a central laboratory that can offer testing services for many regions. With the accumulation of large data volumes from different areas, more comprehensive and accurate results can be achieved.

## Figures and Tables

**Table 1:** Calculation of the prior probability of each array.

Repeat counts	Loci							
	MTUB21	MTUB04	QUB-18	QUB-26	QUB-11b	MIRU31	MIRU10	MIRU26
1								
2	*O*_1,2_							
3		*O*_2,3_						
4			*O*_3,4_	*O*_4,4_				*O*_8,4_
5					*O*_5,5_			
6							*O*_7,6_	
7								
8						*O*_6,8_		
9								
10								

**Table 2:** Prior distribution of two test results.

Repeated number		MTUB21	MTUB04	QUB-18	QUB-26	QUB-11b	MIRU31	MIRU10	MIRU26
0	1st month	0.00	0.00	0.03	0.00	0.00	0.00	0.00	0.00
	2nd month	0.00	0.00	0.05	0.00	0.00	0.00	0.00	0.00
	Difference	0.00	0.00	−0.01	0.00	0.00	0.00	0.00	0.00

1	1st month	0.04	0.01	0.00	0.00	0.01	0.00	0.00	0.01
	2nd month	0.07	0.01	0.01	0.02	0.02	0.01	0.01	0.01
	Difference	−0.02^*∗*^	0.00	−0.01	−0.02	−0.01	−0.01	−0.01	0.00

2	1st month	0.03	0.09	0.03	0.03	0.02	0.01	0.16	0.01
	2nd month	0.04	0.08	0.03	0.03	0.01	0.03	0.15	0.02
	Difference	−0.01	0.01	0.01	0.01	0.01	−0.02	0.01	−0.01

3	1st month	0.07	0.09	0.04	0.02	0.07	0.10	0.76	0.04
	2nd month	0.08	0.18	0.04	0.03	0.09	0.08	0.74	0.03
	Difference	−0.02	−0.09	0.00	−0.01	−0.02	0.02	0.02	0.01

4	1st month	0.18	0.70	0.03	0.13	0.10	0.05	0.05	0.08
	2nd month	0.20	0.63	0.02	0.08	0.11	0.08	0.07	0.06
	Difference	−0.01	0.07	0.01	0.05	−0.02	−0.03	−0.01	0.02

5	1st month	0.52	0.12	0.07	0.04	0.21	0.67	0.01	0.09
	2nd month	0.49	0.11	0.07	0.04	0.21	0.68	0.02	0.07
	Difference	0.03	0.01	−0.01	0.00	0.00	0.00	−0.01	0.02

6	1st month	0.07	0.00	0.03	0.09	0.49	0.08	0.01	0.04
	2nd month	0.05	0.00	0.02	0.08	0.41	0.07	0.01	0.07
	Difference	0.01	0.00	0.01	0.01	0.08	0.01	0.00	−0.02

7	1st month	0.01	0.00	0.05	0.13	0.10	0.01	0.00	0.08
	2nd month	0.01	0.00	0.06	0.16	0.14	0.01	0.00	0.08
	Difference	0.00	0.00	−0.01	−0.02	−0.04	0.00	0.00	0.00

8	1st month	0.04	0.00	0.34	0.41	0.01	0.01	0.00	0.50
	2nd month	0.03	0.00	0.36	0.43	0.01	0.01	0.00	0.50
	Difference	0.01	0.00	−0.03	−0.02	0.00	0.00	0.00	0.00

9	1st month	0.03	0.00	0.18	0.12	0.00	0.00	0.00	0.14
	2nd month	0.03	0.00	0.17	0.11	0.00	0.00	0.00	0.14
	Difference	0.01	0.00	0.02	0.01	0.00	0.00	0.00	0.00

10	1st month	0.00	0.00	0.14	0.02	0.00	0.07	0.00	0.01
	2nd month	0.00	0.00	0.14	0.03	0.00	0.04	0.00	0.02
	Difference	0.00	0.00	0.01	−0.01	0.00	0.02	0.00	−0.01

11	1st month	0.00	0.00	0.03	0.00	0.00	0.00	0.00	0.00
	2nd month	0.00	0.00	0.03	0.00	0.00	0.00	0.00	0.00
	Difference	0.00	0.00	0.01	0.00	0.00	0.00	0.00	0.00

12	1st month	0.00	0.00	0.01	0.00	0.00	0.00	0.00	0.00
	2nd month	0.00	0.00	0.01	0.00	0.00	0.00	0.00	0.00
	Difference	0.00	0.00	0.00	0.00	0.00	0.00	0.00	0.00

^*∗*^The minus sign means the result of the sample collected in first month minus the result of the sample collected in the second.

## Data Availability

The TB type data used to support the findings of this study are available from the corresponding author upon request.
